# Use of Running Equipment Predicts Running-Related Injury in Adult Runners: A Cohort Study of 7347 Runners From the Garmin-RUNSAFE Running Health Study

**DOI:** 10.1155/tsm2/6630254

**Published:** 2025-08-15

**Authors:** Mathias Kristian Pedersen, Frederikke Vestergaard Rasmussen, Ida Lindman, Josefin Abrahamson, Rasmus Østergaard Nielsen

**Affiliations:** ^1^Department of Public Health, Aarhus University, Aarhus, Denmark; ^2^General Practice/Family Medicine, School of Public Health and Community Medicine, Institute of Medicine, University of Gothenburg, Sahlgrenska Academy, Gothenburg, Sweden; ^3^Research, Education, Development & Innovation, Primary Health Care, Gothenburg, Region Västra Götaland, Sweden; ^4^Orthopaedic Research Unit, Sahlgrenska University Hospital, Gothenburg, Sweden; ^5^Department of Health and Rehabilitation, Institute of Neuroscience and Physiology at Sahlgrenska Academy, University of Gothenburg, Gothenburg, Sweden; ^6^Research Unit for General Practice, Aarhus, Denmark

## Abstract

**Background:** Running-related injuries are the most common reason to quit running. There is a gap of knowledge on the use of running equipment as a predictor of running-related injuries in running populations.

**Objective:** To investigate if runners not using equipment while running have a higher rate of running-related injuries compared with runners using running equipment.

**Design and Methods:** Cohort study with an 18-month follow-up, including runners > 18 years old. Exposure was defined as running equipment use, grouped by specific equipment: ankle or knee braces, - tape, insoles, compression socks, jogging stroller, and backpack. Additional groups included participants using multiple types of equipment, those unsure about their equipment use, and those who both indicated uncertainty and selected equipment.

**Results:** Of the 7347 runners included, 3713 (51%) sustained a running-related injury. Runners using knee braces (hazard ratio [HR] = 1.48 [95% CI: 1.14–1.92]), ankle tape (HR = 2.10 [95% CI: 1.44–3.07]), knee tape (HR = 1.83 [95% CI: 1.19–2.82]), insoles (HR = 1.34 [95% CI: 1.20–1.49]), compression socks (HR = 1.14 [95% CI: 1.02–1.27]), or multiple types of equipment (HR = 1.27 [95% CI: 1.15–1.40]) were more prone to injury, while runners using a jogging stroller were less prone (HR = 0.63 [95% CI: 0.39–0.99]). No significant associations were observed for ankle braces (HR = 1.52 [95% CI: 0.90–2.58]), backpacks (HR = 1.00 [95% CI: 0.87–1.14]), runners uncertain about equipment (HR = 0.60 [95% CI: 0.25–1.44]), and uncertain runners who still selected equipment (HR = 1.01 [95% CI: 0.61–1.67]).

**Conclusion:** Runners using certain types of running equipment (e.g., compression socks, insoles, and/or knee brace) while running had higher hazard rates for running-related injuries compared with runners not using running equipment. Owing to the predictive nature of the study, no causal claims between equipment use and running-related injuries can be made.

## 1. Introduction

Physical activity is essential for maintaining health. It is widely suggested that it prevents premature mortality and various chronic diseases [1, 2]. Running is a widely practiced form of physical activity worldwide, as it is an accessible exercise that can be performed in diverse environments and generally requires only a pair of shoes [3]. Although running may be associated with health benefits, it also carries certain risks, namely, running-related injuries (RRIs) [4].

RRIs are considered one of the primary reasons runners discontinue running [5, 6]. The incidence of RRI has been investigated across different populations and is estimated to range from 2.5 to 33.0 injuries per 1000 h of running [4]. Individuals with limited or no running experience [4, 7–9], a high body mass index (BMI) [8, 10, 11], or a history of previous injury [11–14] have increased risks of RRIs. The duration of RRIs can be weeks to months, depending on the injury type [15, 16]. For injured runners, RRIs are associated with stress and anxiety, particularly when daily activities are affected or the ability to run is impeded [17, 18].

To mitigate the risk of RRIs, runners often employ different types of running equipment such as insoles, knee and ankle braces, or sports tape, to protect and support vulnerable areas of the body [19–21]. A previous study of Dutch runners found 19% of the included runners utilized insoles to prevent RRIs during recreational running [22]. Nevertheless, there is still a lack of knowledge regarding the use of different types of running equipment within the general population. Despite growing interest in the relationship between running equipment use and RRIs, the existing body of research remains limited and inconclusive [23]. Studies in this area involve diverse populations, exhibit methodological limitations, or focus primarily on the role of running equipment in treating RRIs rather than preventing them [23]. For instance, some research suggests that certain types of equipment, such as knee braces or custom-made insoles, may help prevent or alleviate specific injuries like knee pain [24, 25], ankle sprains [26], and shin splints [27, 28]. However, the findings often vary due to differences in study design, participant groups, and the types of injuries addressed.

Studies with a predictive purpose that can identify groups of runners at increased risk for RRIs are warranted. To our knowledge, no studies have examined whether runners using different types of equipment, or multiple-use of equipment, have a lower risk of RRIs compared with runners not using any running equipment. Therefore, the purpose of the present study was to investigate if runners above 18 years old who, at baseline, reported not using running equipment have higher risk of RRIs compared with runners using running equipment while running.

## 2. Materials and Methods

### 2.1. Design

This study was designed as a cohort study with an 18-month follow-up period. Data were obtained from the Garmin-RUNSAFE Running Health Study, which has been described in detail (including the questionnaires used) elsewhere [29]. Participants were recruited from mid-2019, with the study terminating at the end of 2020. The study received approval from the Danish Data Protection Agency (notification number: 2015-57-0002). Data handling adhered to applicable regulations under the Personal Data Act. Approval from the Scientific Ethics Committee for the Central Denmark Region was submitted prior to data collection (notification number: 1-10-72-189-16). Exemption from formal review was granted due to the observational nature of the study, as approval is not legally required in Denmark for such studies. All participants provided informed written consent prior to inclusion in the study and retained the right to withdraw their consent at any time. Data management was conducted on a secure server via a virtual desktop interface (VDI) solution at Aarhus University, Denmark, ensuring compliant storage and processing of personally identifiable, subjective, and objective data in accordance with the Danish Data Protection Agency's GDPR. No preregistration of the analysis protocol was made for the present study. However, the statistical coding has been shared in the supporting information (available here).

### 2.2. Participants/Study Population

Participants from the Garmin-RUNSAFE study were included if they met the following criteria: over 18 years of age, proficient in English, regularly uploaded their running activities to Garmin Connect, were willing to complete recruitment, baseline, weekly, monthly, and quarterly questionnaires, agreed to provide access to their Garmin Connect data, and provided informed consent to participate in the study. Exclusion criteria were participants who had a Garmin Connect account or Garmin watch used by more than one person, or if the same running activity was uploaded multiple times to Garmin Connect. Runners did not receive any incentives for participation.

### 2.3. Data Description

#### 2.3.1. Exposure

The use of running equipment was the main exposure in this study. Information on the use of running equipment was self-reported through the following question in a baseline questionnaire: “Do you currently use one or more of the following items when running?” Participants were categorized into separate groups based on the use of individual types of equipment: ankle brace, knee brace, ankle tape, knee tape, insoles, compression socks, jogging stroller, and backpack. Three additional groups were created for participants using multiple types of equipment, for those who indicated “I do not know” regarding their use of running equipment, and for participants who both selected “I do not know” and simultaneously chose a type of running equipment. The group not using running equipment included participants who indicated “No, I do not use any equipment listed above” for the same question.

#### 2.3.2. Outcome

Incidence of RRI was the outcome of the study. An RRI was defined as “musculoskeletal pain in the lower extremities that limits or prevents running (distance, pace, time, or training session) for at least 7 days or three consecutive training sessions, or that requires consultation with a physician or other healthcare professional” [30]. Data on injury status were self-reported based on the following question in the weekly questionnaire: “In the past week, have you had a musculoskeletal injury, or have you experienced a problem to muscles, tendons, or bones that is fully or partly caused by running?” Participants who answered “Yes, I have had an injury” were classified as having had an RRI, whereas participants reporting “No” or “Yes, I have had a problem” were considered injury-free. The questions in the weekly questionnaire regarding injury status are based on the Oslo Trauma Research Center Questionnaire (OSTRC-Q), which is a validated measurement tool for collecting data on overuse injuries [31].

### 2.4. Statistical Methods

Descriptive statistics were used to summarize relevant information about participants' age, gender, BMI, cumulative running distance, incidence of RRI, previous injury, and running experience. Histograms and quantile–quantile plots were used to test for normality. Parametric continuous data were reported as means with standard deviations (SDs), and otherwise as median with interquartile range (IQR). Categorical data were reported as frequencies and percentages. Time to RRI was analyzed using Cox regression model with cumulative running distance in kilometers during follow-up as the time scale. Participants were censored in case of illness, 6 months of nonregistration of running activities on Garmin Connect, withdrawal from the study, or at the end of follow-up on December 31, 2020, whichever came first. Additionally, participants were censored if they failed to respond to four consecutive weekly questionnaires. The assumption of proportional hazards across the exposure groups was evaluated, and proportionality was observed between runners using knee brace, ankle tape, knee tape, insoles, and multi-use of equipment and those not using equipment. All statistical analyses were made using R Studio (2024 Posit Software, PBC, formerly RStudio, PBC), an integrated development environment for the R programming language (2023, the R Foundation for Statistical Computing). Data availability is not available due to ehtical reasons. The statistical analyses can be found in the supporting information.

## 3. Results

**Figure 1:**
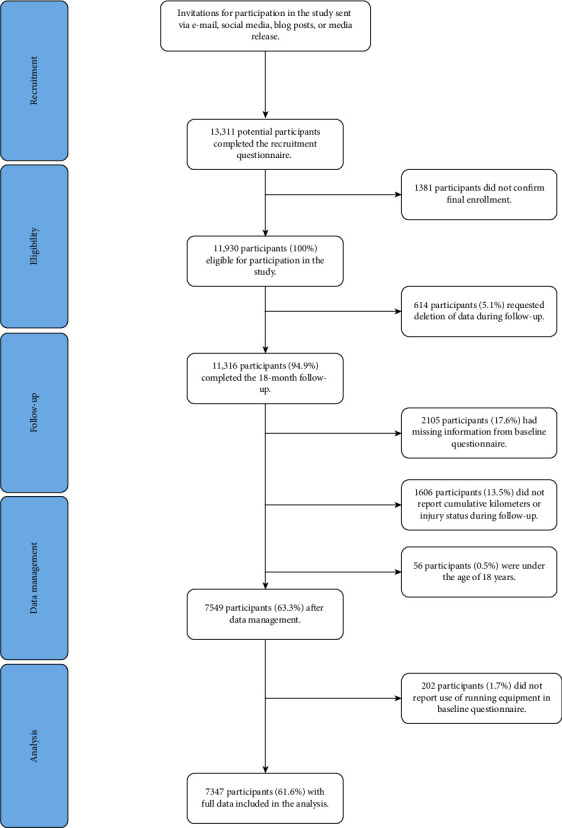
Flow of participants in the study.

A total of 13,311 individuals from 87 different countries responded to the study invitation and completed the recruitment questionnaire. The final number used for analysis was 7347 runners, who collectively ran 4,484,535 km until RRI, censoring, or the end of follow-up on December 31, 2020. The flow of participants in the study is presented in Figure 1.

Participants' characteristics are presented in Table 1. The group of runners was predominantly male (78%) and had a mean age of 46.4 (SD 10.5) years, and nearly half had more than 10 years of running experience (46%). In total, 3713 participants (51%) had an RRI during the study's follow-up period.

The main results from the Cox regression are shown in Table 2. Statistically significant hazard ratios (HRs) for RRI were observed among runners using knee braces (HR = 1.48 [95% CI: 1.14–1.92]), ankle tape (HR = 2.10 [95% CI: 1.44–3.07]), knee tape (HR = 1.83 [95% CI: 1.19–2.82]), insoles (HR = 1.34 [95% CI: 1.20–1.49]), compression socks (HR = 1.14 [95% CI: 1.02–1.27]), jogging strollers (HR = 0.63 [95% CI: 0.39–0.99]), and multi-use of equipment (HR = 1.27 [95% CI: 1.15–1.40]) compared to runners without equipment.

## 4. Discussion

This study found that runners using different types of running equipment had a statistically significant higher rate of sustaining RRIs compared with runners not using running equipment. Specifically, the injury rate was 14%–110% higher compared with those not using such equipment. In contrast, runners running with a jogging stroller had a 37% lower rate of RRIs compared with runners not using any running equipment.

Previous studies have investigated the association between the use of running equipment and risk of an RRI. For instance, Wen et al. [32] found that runners using insoles had nearly double the odds of RRIs compared with those not using insoles (OR = 1.98; *p*=0.048). Furthermore, McKean et al. [33] reported higher odds of RRI among runners using insoles both for runners below 40 years (OR = 1.91; [95% CI: 1.47; 2.49]; *p* < 0.001) and over 40 years (OR = 1.83; [95% CI: 1.33; 2.53]; *p* < 0.001). Chang et al. [34] found that runners using knee braces had double the odds of knee pain (OR = 2.01; [95% CI: 1.31; 3.11]; *p*=0.002), and those using ankle braces had more than triple the odds of ankle pain (OR = 3.49; [95% CI: 1.41; 8.63]; *p*=0.007). These findings align well with the results from the current study.

Due to the observational nature of the present study, it is not possible to draw definitive causal conclusions regarding the relationship between the use of running equipment and the risk of RRI. As seen in Table 1, a larger proportion of runners using equipment have had previous problems related to running. This suggests that runners who use equipment may already be experiencing RRIs and are using the equipment in an effort to prevent additional injuries. Instead of concluding that equipment like insoles, knee braces, and ankle supports leads to injury risk, it may be that the real risk factor is a combination of previous injury history and excessive training load, which together contribute to the likelihood of injury [35]. One can only speculate why runners who run with jogging strollers had 37% lower risk of RRIs compared with runners without equipment. One explanation might be that runners with a jogging stroller have a hard time progressing their running distance and/or pace in an excessive manner as the child needs attention and care [35].

### 4.1. Strengths and Limitations

A strength of the present study is the inclusion of a large cohort of international runners from various countries and skill levels. Previous studies often included smaller cohorts from specific countries or runners of particular levels (e.g., beginners or marathon runners), leading to context-specific results not always applicable to the general running population. The cohort in this study reflects a diverse range of runners, making the results relevant for both novice and elite runners. The possibility of choosing between different types of equipment and multi-use of equipment is another strength, as studies typically investigate one type of equipment. A further strength is the adherence to the study's predictive aim. Conflation between causality and prediction is common in studies with predictive purposes [36]. The aim was to describe which runners were at an increased immediate risk for an RRI, not to explain why they were at increased risk.

However, there are limitations needed to be addressed. It is possible that runners more prone to injuries chose to participate in a study investigating RRI, with over half (54%) reporting a running-related issue in the 3 months before baseline causing a possible selection bias. The dropout of 3297 runners during the follow-up could be related to the outcome if runners who experienced RRI dropped out without reporting it. However, dropout was not assumed to be related to the exposure. It is unlikely that runners with and without equipment systematically differed in their likelihood of dropping out without reporting RRI, thus minimizing potential selection bias from these dropouts. Information on running equipment was self-reported at baseline, leading to potential misclassification if participants changed their equipment use during the study. Such misclassifications are assumed to be nondifferential with respect to the outcome, likely resulting in HR estimates closer to 1.0 than they should be. Injury status was collected through weekly questionnaires. Misclassification could occur if runners incorrectly reported RRI as a running-related problem or vice versa. To avoid this, runners were informed about the difference in the questionnaire.

## 5. Conclusion

This study demonstrated that runners using certain types of running equipment had an increased rate for RRI compared with those not using any running equipment. Owing to the predictive nature of the study, no causal claims between equipment use and RRIs can be made.

## Figures and Tables

**Table 1:** Participant characteristics among included runners distributed across exposure groups based on use of running equipment.

Variable	Total *n* = 7347	No equipment *n* = 4285	Ankle brace *n* = 29	Knee brace *n* = 114	Ankle tape *n* = 38	Knee tape *n* = 38	Insoles *n* = 697	Compression socks *n* = 737	Jogging stroller *n* = 57	Backpack *n* = 457	Multi-use *n* = 836	Uncertain *n* = 20	Uncertain + equipment *n* = 39
Mean age (SD), years^1^	46.4 (10.5)	46.4 (10.6)	45.2 (11.3)	48.4 (10.5)	48.2 (10.2)	47.7 (11.7)	49.4 (11.0)	45.7 (10.0)	37.2 (4.5)	43.9 (9.4)	45.7 (10.1)	45.7 (10.9)	44.2 (9.2)
Gender, *n* (%)													
Female	1636 (22%)	903 (21%)	13 (45%)	25 (22%)	11 (29%)	12 (32%)	186 (27%)	142 (19%)	19 (33%)	100 (22%)	219 (26%)	2 (10%)	4 (10%)
Male	5711 (78%)	3382 (79%)	16 (55%)	89 (78%)	27 (71%)	26 (68%)	511 (73%)	595 (81%)	38 (67%)	357 (78%)	617 (74%)	18 (90%)	35 (90%)
Median BMI (IQR), kg/m^2^	24.06 (4.29)	24.02 (4.19)	24.77 (6.28)	24.85 (4.78)	24.24 (4.78)	24.57 (4.98)	23.95 (4.20)	24.22 (4.43)	22.91 (3.63)	23.84 (4.28)	24.13 (4.44)	23.21 (5.77)	24.34 (5.30)
Median cumulative kilometers (IQR)	238.76 (715.2)	255.11 (742.3)	68.42 (384.8)	96.80 (464.3)	120.19 (387.6)	98.57 (292.5)	217.06 (645.1)	204.54 (632.9)	404.82 (836.6)	417.14 (910.2)	196.06 (620.5)	261.38 (609.9)	157.08 (567.0)
Injury, *n* (%)	3713 (51%)	2048 (48%)	14 (48%)	59 (52%)	27 (71%)	21 (55%)	418 (60%)	375 (51%)	18 (32%)	250 (55%)	463 (55%)	5 (25%)	15 (38%)
Running experience, *n* (%)^2^													
0–5 years	1951 (27%)	1121 (26%)	6 (21%)	39 (35%)	9 (24%)	12 (32%)	142 (21%)	234 (32%)	11 (19%)	129 (28%)	228 (28%)	8 (40%)	12 (32%)
5–10 years	1915 (26%)	1129 (27%)	6 (21%)	24 (21%)	10 (26%)	12 (32%)	147 (21%)	189 (26%)	13 (23%)	138 (30%)	231 (28%)	6 (30%)	10 (26%)
More than 10 years	3428 (47%)	2009 (47%)	16 (58%)	49 (44%)	19 (50%)	14 (36%)	401 (58%)	309 (42%)	33 (58%)	189 (42%)	367 (44%)	6 (30%)	16 (42%)
Previous problem, *n* (%)^3^													
No previous problem	3374 (46%)	2218 (52%)	9 (31%)	30 (27%)	2 (5%)	8 (21%)	265 (38%)	295 (40%)	29 (51%)	216 (48%)	278 (33%)	8 (40%)	16 (43%)
Yes	3934 (54%)	2047 (48%)	20 (69%)	83 (73%)	36 (95%)	30 (79%)	429 (62%)	436 (60%)	28 (49%)	238 (52%)	654 (67%)	12 (60%)	21 (57%)

Abbreviations: IQR = interquartile range, *n* = number, and SD = standard deviation.

^1^Missing values: total—12, no equipment—7, ankle brace—1, insoles—1, and multi-use—3.

^2^Missing values: total—53, no equipment—26, ankle brace—1, knee brace—2, insoles—7, compression socks—5, backpack—1, multi-use—10, and uncertain + equipment—1.

^3^Missing values: total—39, no equipment—20, knee brace—1, insoles—3, compression socks—6, backpack—3, multi-use—4, and uncertain + equipment—2.

**Table 2:** Cox regression model for the association between types of running equipment and RRI (*N* = 7347, number of runners with an RRI = 3713).

Types of running equipment	HR	95% CI	*p*
No use of equipment	1.00	—	—
Ankle brace	1.52	[0.90; 2.58]	0.117
Knee brace	1.48	[1.14; 1.92]	0.003
Ankle tape	2.10	[1.44; 3.07]	< 0.001
Knee tape	1.83	[1.19; 2.82]	0.006
Insoles	1.34	[1.20; 1.49]	< 0.001
Compression socks	1.14	[1.02; 1.27]	0.019
Jogging stroller	0.63	[0.39; 0.99]	0.047
Backpack	1.00	[0.87; 1.14]	0.954
Multi-use (> 1 equipment)	1.27	[1.15; 1.40]	< 0.001
Uncertain	0.60	[0.25; 1.44]	0.250
Uncertain + equipment	1.01	[0.61; 1.67]	0.978

Abbreviations: 95% CI, 95^th^ percent confidence interval; HR, hazard ratio; RRI, running-related injury.

## Data Availability

Data are not available due to ethical reasons.

## Supporting Information

The supporting information includes statistical analyses.
