# Supplementary material for: Use of Running Equipment Predicts Running-Related Injury in Adult Runners: A Cohort Study of 7347 Runners From the Garmin-RUNSAFE Running Health Study
Source: Transl Sports Med. 2025 Aug 15;2025:6630254. doi: 10.1155/tsm2/6630254 (PMC12373465; doi:10.1155/tsm2/6630254)
Supplement: Supporting Information — Additional supporting information can be found online in the Supporting Information section. [file 6630254.f1.docx]

### PREFACE

# This code shows the data management and statistical analysis used for the

# present study. Participants have been censored in case of illness, six months

# of non-registration of running activities on Garmin Connect, withdrawal from

# the study, or at the end of follow-up, whichever came first.

# The following variables are assumed to be present in the data set:

# - id: id of participants

# - record: the number of running sessions completed during follow-up

# - injury: whether or not the runner sustained an injury (true/false)

# - cumkm: the cumulative running distance in kilometres for each runnner

# - age: age of runner

# - gender: sex of runner (male/female)

# - BMI: BMI of runner

# - equip_b: sequence of numbers corresponding to runner's use of equipment from

# the baseline questionnaire. A unique sequence is generated for each possible

# variation of equipment usage.

# - prevprob: whether or not the runner reported a previous running-related

# problem (true/false)

# - experience: runner's experience in years

### DATA MANAGEMENT

# Load data

runsafe <- read.csv("Data 22042024.csv")

# Generate BMI variable

runsafe <- mutate(runsafe, height_m = height_cm / 100)

runsafe <- mutate(runsafe, BMI = weight_kg / height_m^2)

# We change missing or erroneous values to NA in the prevprob variable

runsafe$prevprob <- ifelse( runsafe$prevprob=="Missing"| runsafe$prevprob=="" | runsafe$prevprob=="2"|runsafe$prevprob=="3", NA, runsafe$prevprob)

# We change missing or erroneous values to NA in the experience variable

runsafe$experience <- ifelse(runsafe$experience == "99" | runsafe$experience=="Missing" |runsafe$experience=="I dont know", NA, runsafe$experience)

# We change missing values to NA in the equipment variable

runsafe$equipment <- ifelse(runsafe$equipment =="Missing"| runsafe$equipment=="", NA, runsafe$equipment)

# We generate a new variable for equipment usage that is more legible

runsafe <- runsafe |> mutate(equipment = case_when(

equip_b == 1000000 ~ "10",

equip_b == 50000000000 ~ "5",

equip_b == 20000000000000 ~ "2",

equip_b == 3000000000000 ~ "3",

equip_b == 6000000000 ~ "6",

equip_b == 80000000 ~ "8",

equip_b == 450000000000 ~ "45",

equip_b == 700000000 ~ "7",

equip_b == 20006000000000 ~ "26",

equip_b == 50080000000 ~ "58",

equip_b == 56080000000 ~ "568",

equip_b == 780000000 ~ "78",

equip_b == 100000000000000 ~ "1",

equip_b == 56000000000 ~ "56",

equip_b == 6080000000 ~ "68",

equip_b == 3006000000000 ~ "36",

equip_b == 56700000000 ~ "567",

equip_b == 50700000000 ~ "57",

equip_b == 103000000000000 ~ "13",

equip_b == 100000080000000 ~ "18",

equip_b == 9000000 ~ "9",

equip_b == 3400000000000 ~ "34",

equip_b == 400000000000 ~ "4",

equip_b == 801000000 ~ "81",

equip_b == 91000000 ~ "91",

equip_b == 6009000000 ~ "69",

equip_b == 67001000000 ~ "671",

equip_b == 6780000000 ~ "678",

equip_b == 500001000000 ~ "51",

equip_b == 3056000000000 ~ "356",

equip_b == 3406080000000 ~ "3468",

equip_b == 20056000000000 ~ "256",

equip_b == 3050000000000 ~ "35",

equip_b == 3006080000000 ~ "368",

equip_b == 20050000000000 ~ "25",

equip_b == 103056080000000 ~ "13568",

equip_b == 3450000000000 ~ "345",

equip_b == 6700000000 ~ "67",

equip_b == 23000000000000 ~ "23",

equip_b == 100050080000000 ~ "158",

equip_b == 103050000000000 ~ "135",

equip_b == 3000080000000 ~ "38",

equip_b == 20400000000000 ~ "24",

equip_b == 100056000000000 ~ "156",

equip_b == 23456000000000 ~ "23456",

equip_b == 20006780000000 ~ "2678",

equip_b == 20006080000000 ~ "268",

equip_b == 100006000000000 ~ "16",

equip_b == 400080000000 ~ "48",

equip_b == 0 ~ "0",

equip_b == 120006000000000 ~ "126",

equip_b == 100050000000000 ~ "15",

equip_b == 406080000000 ~ "468",

equip_b == 456000000000 ~ "456",

equip_b == 406000000000 ~ "46",

equip_b == 23056080000000 ~ "23568",

equip_b == 103050080000000 ~ "1358",

equip_b == 20456080000000 ~ "24568",

equip_b == 100006080000000 ~ "168",

equip_b == 100050700000000 ~ "157",

equip_b == 60001000000 ~ "61",

equip_b == 20450000000000 ~ "245",

equip_b == 20006700000000 ~ "267",

equip_b == 23406000000000 ~ "2346",

equip_b == 120406080000000 ~ "12468",

equip_b == 20406000000000 ~ "246",

equip_b == 123000000000000 ~ "123",

equip_b == 103006000000000 ~ "136",

equip_b == 20000700000000 ~ "27",

equip_b == 20056080000000 ~ "2568",

equip_b == 100400000000000 ~ "14",

equip_b == 20050080000000 ~ "258",

equip_b == 103406000000000 ~ "1346",

equip_b == 20456000000000 ~ "2456",

equip_b == 3006089000000 ~ "3689",

equip_b == 100056080000000 ~ "1568",

equip_b == 3456000000000 ~ "3456",

equip_b == 7001000000 ~ "71",

equip_b == 123006000000000 ~ "1236",

equip_b == 23006000000000 ~ "236",

equip_b == 3006700000000 ~ "367",

equip_b == 120000000000000 ~ "12",

equip_b == 450080000000 ~ "458",

equip_b == 120000080000000 ~ "128",

equip_b == 60091000000 ~ "691",

equip_b == 120006080000000 ~ "1268",

equip_b == 456080000000 ~ "4568",

equip_b == 406780000000 ~ "4678",

equip_b == 20400080000000 ~ "248",

equip_b == 60801000000 ~ "681",

equip_b == 3406000000000 ~ "346",

equip_b == 23400000000000 ~ "234",

equip_b == 20000080000000 ~ "28",

equip_b == 23006080000000 ~ "2368",

equip_b == 3056080000000 ~ "3568",

equip_b == 120056000000000 ~ "1256",

equip_b == 400700000000 ~ "47",

equip_b == 50780000000 ~ "578",

equip_b == 450700000000 ~ "457",

equip_b == 123050080000000 ~ "12358",

equip_b == 20450080000000 ~ "2458",

equip_b == 67801000000 ~ "6781",))

# We categorize the numerical values of the new variable to align with the

# corresponding categories of equipment usage among the runners

runsafe <- runsafe |> mutate(

equipmentgrp = case_when(

equipment %in% c(1) ~ "Ankle brace",

equipment %in% c(2) ~ "Knee brace",

equipment %in% c(3) ~ "Ankle tape",

equipment %in% c(4) ~ "Knee tape",

equipment %in% c(5) ~ "Insoles",

equipment %in% c(6) ~ "Compression socks",

equipment %in% c(7) ~ "Baby jogger",

equipment %in% c(8) ~ "Backpack",

equipment %in% c(9) ~ "I dont know",

equipment %in% c(81,91,69,671,51,0,61,3689,71,691,681, 6781) ~ "I dont know + other equipment",

equipment %in% c(78,68,678,67,45,35,34,25,345,234,23,12,13,135,24,15,245, 123,14,56,26,58,568,36,18,256,38,156,16,48,46,28,567,57,356,3468,368,13568,158,13456,2678,268,126,468,456,23568,1358,24568,168,157,267,2346,12468,246,136,27,2568,258,1346,2456,1568,3456,1236,236,367,458,128,1268,4568,4678,248,346,234,28,2368,3568,1256,47,578,457,12358,2458,23456) ~ "Multi-use",

equipment %in% c(10) ~ "No Equipment",))

# We reduce the number of categories in the experience variable

runsafe <- runsafe |> mutate(experience_grp = case_when(

experience == "Below 1 year" ~ "0-5 years",

experience == "1-3 years" ~ "0-5 years",

experience == "3-5 years" ~ "0-5 years",

experience == "5-10 years" ~ "5-10 years",

experience == "10-20 years" ~ "More than 10 years",

experience == "20-40 years" ~ "More than 10 years",

experience == "More than 40 years" ~ "More than 10 years"

))

runsafe <- runsafe |> mutate(

experience_grp = factor(experience_grp),

experience_grp = fct_relevel(

experience_grp, "0-5 years", "5-10 years", "More than 10 years"))

# Remove runners under the age of 18 years. This was done manually from the

# data frame by sorting the runners by age.

runsafe <- runsafe |> filter(

!row_number() %in% c(1164,6353,4965,6253,2706,7326,2879,2535,130,6986,1905,

1728,7250,663,2454,2819,2515,6748,4761,6927,4329,5207,

5124,5212,6598,6767,1851,3129,3223,7173,7487,6428,6805,

7586,7169,3684,5081,1956,3604,7362,236,1886,2899,2720,

4392,3753,4028,5376,2979,5603,3290,2972,6161,2472,

3325,4150))

# Set the reference level for equipment variable and previous problem variable

runsafe <- mutate(runsafe,

equipmentgrp = factor(equipmentgrp),

equipmentgrp = fct_relevel(equipmentgrp, "No Equipment", "Ankle brace",

"Knee brace", "Ankle tape", "Knee tape",

"Insoles", "Compression socks", "Baby jogger",

"Backpack", "Multi-use", "I dont know",

"I dont know + other equipment"),

prevprob = factor(prevprob),

prevprob = fct_relevel(prevprob, "No previous problem"))

# Remove runners with missing values on injury and cumulative distance

runsafe <- runsafe |> filter(!(is.na(injury & cumkm)))

# Remove runners with missing values on equipment usage

runsafe <- runsafe |> filter(!(is.na(equipment)))

### TABLE 1 DEMOGRAPHICS

# We select variables for demographics

demographics <- runsafe |> select(

age, gender, height_m, weight_kg, BMI, cumkm,

injury,experience_grp, prevprob, equipmentgrp)

# We generate and design the layout of the table

demographics |> tbl_summary(by = equipmentgrp,

label = list(

age ~ "Age, years",

gender ~ "Gender",

injury ~ "Injury",

cumkm ~ "Cumulative kilometers",

BMI ~ "BMI, kg/m\u00B2", sep = "\n",

prevprob ~ "Previous Problem",

experience_grp ~ "Running Experience",

height_m ~ "Height, meters",

weight_kg ~ "Weight, kilograms"),

statistic = list(

age = "{mean} ({sd})",

BMI = "{median} ({IQR})",

cumkm = "{median} ({IQR})",

height_m = "{mean} ({sd})",

weight_kg = "{median} ({IQR})",

all_categorical() ~ "{n} / {N} ({p}%)"

),

digits = all_continuous() ~ 2) |>

add_overall() |> add_stat_label() |>

modify_header(label ~ "**Variable**") |>

modify_spanning_header(c(

"stat_1", "stat_2", "stat_3", "stat_4")

~ "**Running Equipment Group**") %>%

modify_caption("**Table 1. Runner Characteristics**") |>

modify_footnote(all_stat_cols() ~ "Mean (SD), Median (IQR) or Frequency (%)"

) |> bold_labels()

### COX REGRESSION

# We change the numerical values to categories for equipment usage

runsafe_cox <- runsafe |> mutate(

equipmentgrp = case_when(

equipment %in% c(1) ~ "Ankle brace",

equipment %in% c(2) ~ "Knee brace",

equipment %in% c(3) ~ "Ankle tape",

equipment %in% c(4) ~ "Knee tape",

equipment %in% c(5) ~ "Insoles",

equipment %in% c(6) ~ "Compression socks",

equipment %in% c(7) ~ "Baby jogger",

equipment %in% c(8) ~ "Backpack",

equipment %in% c(9) ~ "I dont know",

equipment %in% c(81,91,69,671,51,0,61,3689,71,691,681,6781) ~ "I dont know + other equipment",

equipment %in% c(78,68,678,67,45,35,34,25,345,234,23,12,13,135, 24,15,245,123,14,56,26,58,568,36,18,256,38,156,

16,48,46,28,567,57,356,3468,368,13568,158,13456,2678,268,126,468,456,23568,1358,24568,168,157,267,2346,12468,246,136,27,2568,258,1346,2456,1568,3456,1236,236,367,458,128,1268,4568,4678,248,346,234,28,2368,3568,1256,47,578,457,12358,2458,23456) ~ "Multi-use",

equipment %in% c(10) ~ "No Equipment"))

# We set the reference level for equipment usage

runsafe_cox <- mutate(

runsafe_cox, equipmentgrp = factor(equipmentgrp),

equipmentgrp = fct_relevel(equipmentgrp, "No Equipment", "Ankle brace", "Knee brace", "Ankle tape", "Knee tape", "Insoles", "Compression socks", "Baby jogger", "Backpack", "Multi-use", "I dont know", "I dont know + other equipment"),

)

# Now we are ready to fit the Cox regression model

cox <- coxph(Surv(cumkm, injury == "1") ~ equipmentgrp, data = runsafe_cox)

summary(cox)

parameters(cox, include_reference = TRUE, exponentiate = TRUE, digits = 2) |> print(select = "minimal")

# We note the number of participants used in the regression model

nobs(cox)

# Closes the script and generates a log-file

log_script("Statistical Analysis 30062025.R")
